# CCR2 and CD44 Promote Inflammatory Cell Recruitment during Fatty Liver Formation in a Lithogenic Diet Fed Mouse Model

**DOI:** 10.1371/journal.pone.0065247

**Published:** 2013-06-07

**Authors:** Charlotte E. Egan, Erin K. Daugherity, Arlin B. Rogers, Delbert S. Abi Abdallah, Eric Y. Denkers, Kirk J. Maurer

**Affiliations:** 1 Department of Microbiology and Immunology, College of Veterinary Medicine, Cornell University, Ithaca, New York, United States of America; 2 Department of Biomedical Sciences, College of Veterinary Medicine, Cornell University, Ithaca, New York, United States of America; 3 Center for Animal Resources and Education, College of Veterinary Medicine, Cornell University, Ithaca, New York, United States of America; 4 Department of Pathology and Laboratory Medicine; University of North Carolina, Chapel Hill, North Carolina, United States of America; Université Libre de Bruxelles, Belgium

## Abstract

Non-alcoholic fatty liver disease (NAFLD) is a common disease with a spectrum of presentations. The current study utilized a lithogenic diet model of NAFLD. The diet was fed to mice that are either resistant (AKR) or susceptible (BALB/c and C57BL/6) to hepatitis followed by molecular and flow cytometric analysis. Following this, a similar approach was taken in congenic mice with specific mutations in immunological genes. The initial study identified a significant and profound increase in multiple ligands for the chemokine receptor CCR2 and an increase in CD44 expression in susceptible C57BL/6 (B6) but not resistant AKR mice. *Ccr2^−/−^* mice were completely protected from hepatitis and *Cd44^−/−^* mice were partially protected. Despite protection from inflammation, both strains displayed similar histological steatosis scores and significant increases in serum liver enzymes. CD45^+^CD44^+^ cells bound to hyaluronic acid (HA) in diet fed B6 mice but not *Cd44^−/−^* or *Ccr2^−/−^* mice. *Ccr2^−/−^* mice displayed a diminished HA binding phenotype most notably in monocytes, and CD8^+^ T-cells. In conclusion, this study demonstrates that absence of CCR2 completely and CD44 partially reduces hepatic leukocyte recruitment. These data also provide evidence that there are multiple redundant CCR2 ligands produced during hepatic lipid accumulation and describes the induction of a strong HA binding phenotype in response to LD feeding in some subsets of leukocytes from susceptible strains.

## Introduction

Non-alcoholic fatty liver disease (NAFLD) is an increasingly common disease of humans affecting approximately 30–40% of individuals in the United States depending on the study population [1], [2]. NAFLD represents a spectrum of hepatic diseases from benign lipid accumulation in the liver to more severe non-alcoholic steatohepatitis (NASH) which may lead to hepatocellular carcinoma and liver failure [3]. Although a variety of factors including high caloric intake, limited physical activity and other co-morbidities such as diabetes are known to predispose individuals to NAFLD the factors which promote progression of NAFLD to NASH are less clear [4].

The roles of chemokine ligands and their associated receptors are well established in the recruitment of immune cells during inflammation. Ovariectomized mice fed high fat diets display worse hepatitis than their intact counterparts and this exacerbation of disease is associated with increased liver expression of CCL2 [5]. Human patients with NASH also display hepatic upregulation of many chemokines and other leukocyte adhesion molecules [6]. Others have noted systemic elevations of some cytokines and chemokines in patients with NASH [7]. Consistent with the findings in human patients, CCR2 inhibition in obese prone mice fed a “Western” diet reduced adipose tissue inflammation and associated comorbidities including hepatic macrophage recruitment [8], .

Previously, we used a lithogenic diet model of NAFLD/NASH to demonstrate that the cell cycle gene *Atm* was critical in responding to lipid-mediated oxidative damage in affected livers. Further, we showed that in mice lacking *Atm*, lithogenic diet induced hepatocyte apoptosis and fibrosis were significantly diminished [10]. The goal of the present study was to exploit the lithogenic diet model to identify hepatic inflammation susceptible and resistant mouse strains and to establish immunological determinants of disease in the liver. We found that C57BL/6 mice are susceptible BALB/c mice are intermediate and AKR mice are resistant to hepatitis. Protection in AKR mice corresponded to a significant reduction in hepatic chemokine production and a decrease in recruitment of CD44^+^CD45^+^ cells. We observed that deletion of CCR2 on B6 background mice completely eliminates hepatic inflammation, stellate cell activation and hepatic fibrosis despite hepatic lipid accumulation and significant elevations in serological markers of hepatocellular damage. Further, we found that progression of disease is impeded in mice lacking the leukocyte adhesion molecule CD44 and we describe an interaction between CD44 and hepatic hyaluronic acid (HA) in this process. Finally, we show that in the absence of CCR2, CD44 mediated HA binding is not induced on CD8^+^ T-cells and monocytes. Collectively, our studies show that CD44 and CCR2 contribute to hepatitis in a model of NAFLD despite histological evidence of steatosis and elevated serological markers of hepatocellular damage.

## Methods

### Animal Use

Male 4–5 week-old mice were purchased (BALB/cJ, AKRJ, C57BL/6J) from The Jackson Laboratory (Bar Harbor, ME) or bred in house (*Cd44^−/−^*, *Ccr2^−/−^*). CCR2 reporter mice were kindly donated by Eric Pamer (Memorial Sloan Kettering) and then bred in house [11]. Mice were housed in an AAALAC accredited facility under barrier conditions, free of known viral and bacterial pathogens. Animals were given irradiated feed and sterile water. Beginning at 8–10 weeks of age mice were either fed a standard rodent diet (SD) (Harlan Teklad 7912) or a completely synthetic lithogenic diet (LD) (1.0% cholesterol, 0.5% cholic acid, 15.8% total lipid Harlan Teklad TD.09237) for 1–4 weeks. At the conclusion of the study mice were euthanized and animals were either bled by cardiac puncture or perfused with sterile PBS and tissues were collected for analysis. For gross morphologic analysis mice were weighed, euthanized by CO_2_ and livers were extracted intact and weighed.

### Ethics Statement

All animal work was done in accordance with PHS guidelines and was approved by Cornell’s institutional animal care and use committee (Protocol # 2009–0010).

### Liver Leukocyte Preparation

Initial studies in B6, AKR and BALB/c mice utilized all of the liver except three 30 mg tissue sections which were used for histopathology and RNA preparation. Subsequent studies in B6 *Ccr2^−/−^* and *Cd44^−/−^* mice utilized the entire liver. Perfused livers were macerated and strained through a sterile stainless steel mesh and tissue residue was diluted in PBS and centrifuged (10 minutes, 1250 rpm). The tissue was then digested with collagenase as described [12] washed with PBS and made into a single cell suspension. Finally, Percoll gradient centrifugation (40%/80%) was performed to further purify the leukocyte fraction.

### Liver Histopathology

Livers were either formalin fixed and processed for standard histopathological analysis or they were flash frozen in optimum cutting temperature (O.C.T.) embedding media (Sakura Finetek, Torrance, CA) for subsequent fluorescence immunohistochemistry. Flash frozen tissues were fixed and permeabilized in acetone, blocked with serum appropriate to the antibody (Jackson Immunoresearch, West Grove, PA) and then labeled with antibodies to targets (ABCAM, Cambridge, MA) followed by secondary fluorochrome conjugated antibodies (Jackson Immunoresearch, West Grove, PA). Standard histological slides were formalin fixed and processed for hematoxylin and eosin (H&E) or pico-sirius red staining and examined by light microscopy [10]. Oil-red-O staining was conducted on formalin fixed or frozen sections and slides were examined by light microscopy. Immunofluorescently stained tissue was analyzed by confocal microscopy (Leica SP5). H&E and pico-sirius red stained tissues were examined by a veterinary pathologist with expertise in animal models of liver disease who was blinded to group identity. Slides were scored for inflammation, steatosis and fibrosis on an ascending 0–4 scale using previously established criteria (0 = none, 1 = mild, 2 = moderate, 3 = severe, 4 = very severe) [10].

### Cell Staining and Flow Cytometry

Leukocyte enriched fractions from Percoll gradient centrifugation were blocked in flow staining buffer (1% BSA, 0.05% NaN_3_ in PBS) with 10% normal mouse serum (Jackson Immunoresearch, West Grove, PA). Cells were then incubated with fluorochrome-conjugated antibodies specific for CD45, CD44, CD11b, CD11c, CD3, CD8, CD4, Ly6G (GR-1), Ly6C, F480, NK1.1, CD49b, B220, and CD19 (Becton Dickson, Franklin Lakes, NJ and eBioscience, San Diego, CA) in flow staining buffer for 30 minutes on ice, washed by centrifugation and fixed in 3% buffered paraformaldehyde. For the Hyaluronic acid (HA) binding assay, cells were incubated with HA-FITC (Santa Cruz Biotechnology, Santa Cruz, CA) for 30 minutes on ice, washed and fixed as above [13]. Total liver preps were gated on FSC/SSC to exclude debris and cell clumps. Cells were gated based upon single staining of CD45^+^ cells and the FSC/SSC gate of the CD45^+^ cells was applied to all samples (Fig. S1). Approximately 20,000–100,000 cells per analyses were acquired on a FACSCalibur (for the initial strain comparison analysis) or FACSCantoII (for all subsequent studies; Becton Dickson, Franklin Lakes, NJ). Acquired data were analyzed with FlowJo software (Treestar, Ashland, OR). Cell numbers were calculated based upon total cell counts after purification and positive staining for the markers described.

### Quantitative PCR

Hepatic RNA was prepared using commercially available tissue RNA isolation kits (Omega bio-tek, Norcross, GA 30071) from the livers of SD fed B6 and AKR mice (n = 3 each) and LD fed B6, AKR and *Ccr2^−/−^* mice (n = 3 each). RNA was converted to cDNA and inflammatory genes were analyzed by semi-quantitative PCR using the commercially available “Inflammatory Cytokines & Receptors PCR Array” (Qiagen Valencia, CA). Gene expression levels were normalized to the internal housekeeping genes (5 housekeeping genes) included on the individual plates and fold changes were acquired by comparing to SD fed AKR or B6 controls using the ΔΔ Ct method [10]. LD or SD fed B6 mice were statistically compared to AKR or *Ccr2* ^−/−^ mice utilizing company supplied software [14].

### Serum Enzyme Analysis

Immediately after euthanasia, blood was collected and placed into microtainers coated with lithium heparin (Becton Dickson, Franklin Lakes, NJ). Tubes were centrifuged and serum was collected. Alanine aminotransferase (ALT), sorbitol dehydrogenase (SDH) and glutamate dehydrogenase (GLDH) where evaluated from individual, non-diluted, fresh samples by a commercial veterinary reference lab (Animal Health Diagnostic Center, Cornell University, Ithaca, NY).

### Statistical Analyses

Data were analyzed by ANOVA or t-test for parametric data, by the Mann-Whitney-test for nonparametric data and by Fisher’s exact test for correlative data using commercially available software (Prism, Graphpad, La Jolla, CA).

## Results

### C57BL/6 Mice are Susceptible, BALB/c Mice are Intermediate and AKR Mice are Resistant to Lithogenic Diet Feeding Induced Hepatitis

**Figure 1:**
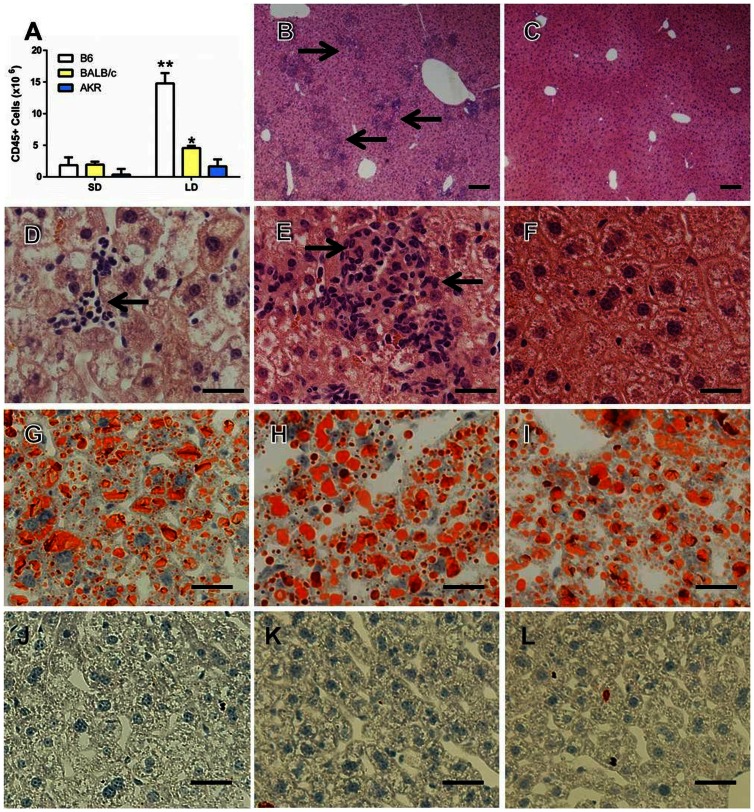
C57BL/6 mice are highly susceptible to diet induced hepatitis. (A) Liver leukocytes (CD45^+^ cells) were quantified in C57BL/6 (B6), BALB/c, and AKR mice fed either a standard diet (SD) or 4 weeks of lithogenic diet (LD) (*P<0.05 compared to SD controls, **P<0.05 compared to all groups; n = 3–5 per group). (B and E) H&E staining of liver from LD fed B6 mice under low and high power magnification respectively. Arrows in B point to inflammatory foci and E shows a representative inflammatory focus. (C and F) H&E staining of liver from LD fed AKR mice under low and high power magnification respectively. Oil-red-O staining showing lipid accumulation in BALB/c (G), B6 (H) and AKR mice (I) fed the LD but not in those fed the SD (J–L). Bars in B and C = 100µm; bars in D–L = 20µm.

**Figure 2:**
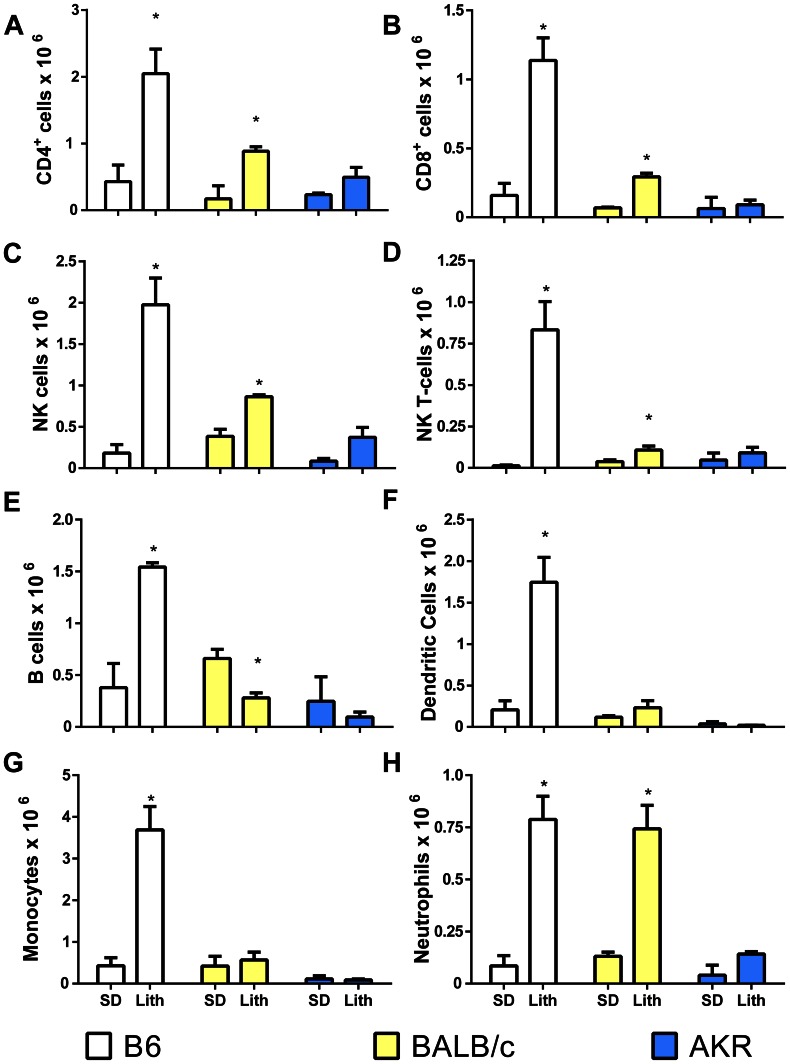
LD fed hepatitis susceptible B6 mice display uniform increases in hepatic leukocyte populations. (A–H) Composition of liver leukocytes in B6, BALB/c, and AKR mice fed SD or LD (*P<0.05 compared to SD controls). Leukocyte populations are defined as follows: NK cells, CD49b^+^ CD3^−^; NK-T cells, CD3^+^ CD49b^+^; B-cells, CD19^+^ B220^+^; dendritic cells, CD11c^+^, macrophages, CD11b^+^ Ly6C^−^, neutrophils, CD11b^+^ Ly6C^+^.

After four weeks of lithogenic diet (LD) feeding C57BL/6 (B6) mice developed hepatitis demonstrated by an approximate 10 fold increase in CD45^+^ inflammatory cells present in the liver when compared to standard diet (SD) fed controls (Fig. 1A, *P<0.05). BALB/c mice fed the same diet also had significant increases in CD45^+^ cells compared to their SD fed counterparts; however, the CD45^+^ cell counts were significantly less than B6 mice fed the same diet. AKR mice did not develop significant increases in CD45^+^ cells compared to their SD fed counterparts and they had significantly fewer liver leukocytes than B6 mice fed the diet (Fig. 1A). Cell number differences between susceptible and resistant mice were confirmed by histological examination (Fig. 1B–F). B6 mice developed moderate to severe multifocal to coalescing hepatitis composed predominantly of mononuclear cells (Fig. 1E arrows). In contrast, BALB/c mice developed only minor focal areas of hepatitis with a more noticeable polymorphonuclear component (Fig. 1D arrows). Inflammatory infiltrates rarely occurred in AKR mice (Fig. 1C, F). Despite striking differences in inflammatory cell infiltration, oil-red-o stained sections of the liver confirmed that susceptible B6, intermediate BALB/c and resistant AKR mouse livers all accumulated excess lipid (Fig. 1 G–I). Consistent with previous publications SD fed mice contained negligible hepatic lipid (Fig. 1 J–L) [10], [15]. Flow cytometric analysis revealed that B6 mice developed increases in all inflammatory cell types examined (Fig. 2).

**Figure 3:**
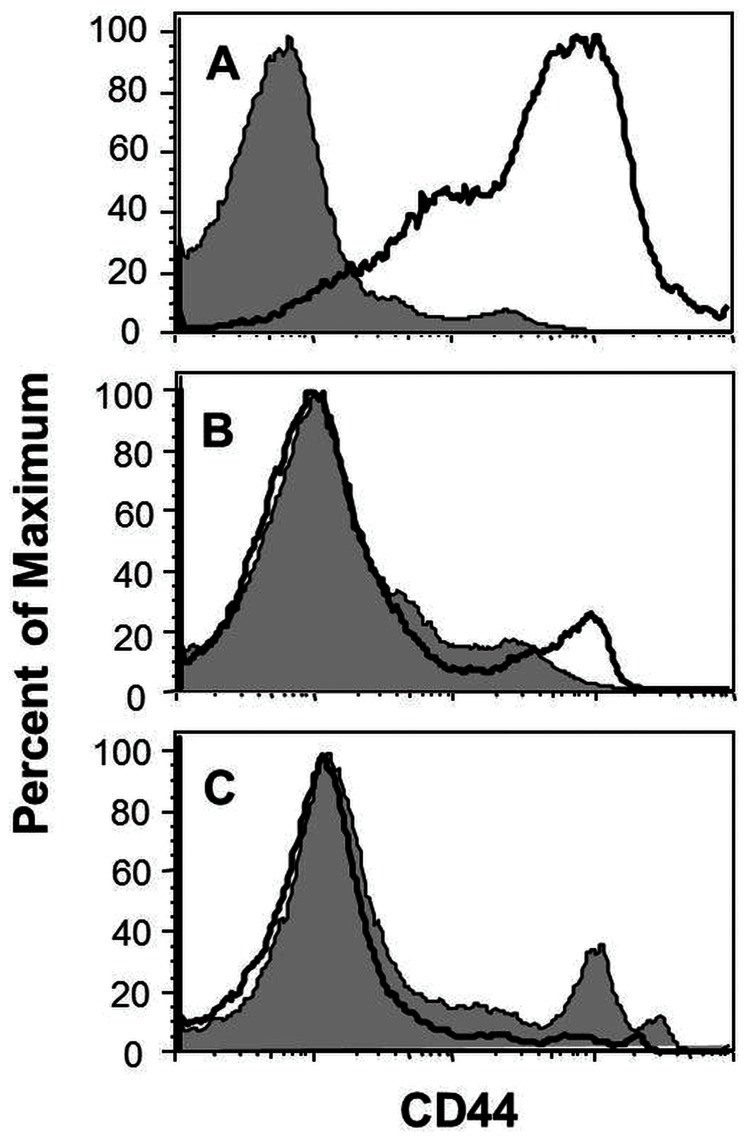
Hepatitis susceptible mouse strains display increased liver leukocyte CD44 expression in response to LD feeding. Hepatic leukocyte preparations were gated on forward and side-scatter and CD44 levels of B6 (A), BALB/c (B) and AKR (C) mice were examined by flow cytometry. Solid lines represent mice on LD and shaded lines represent mice on SD.

We next examined hepatic leukocyte preparations for CD44 expression because this molecule is upregulated in NASH patients and is important in leukocyte recruitment to hepatic sinusoids [6], [16]. We found that CD44 expression on Percoll enriched leukocyte preparations was markedly increased in susceptible B6 mice (Fig. 3A) and moderately increased in BALB/c mice (Fig. 3B) after 4 weeks of LD feeding. In contrast, resistant AKR mice did not display increased expression of CD44 after LD feeding (Fig. 3C).

### Susceptible B6 Mice Develop a Striking Proinflammatory Cytokine Signature after Lithogenic Feeding

**Figure 4:**
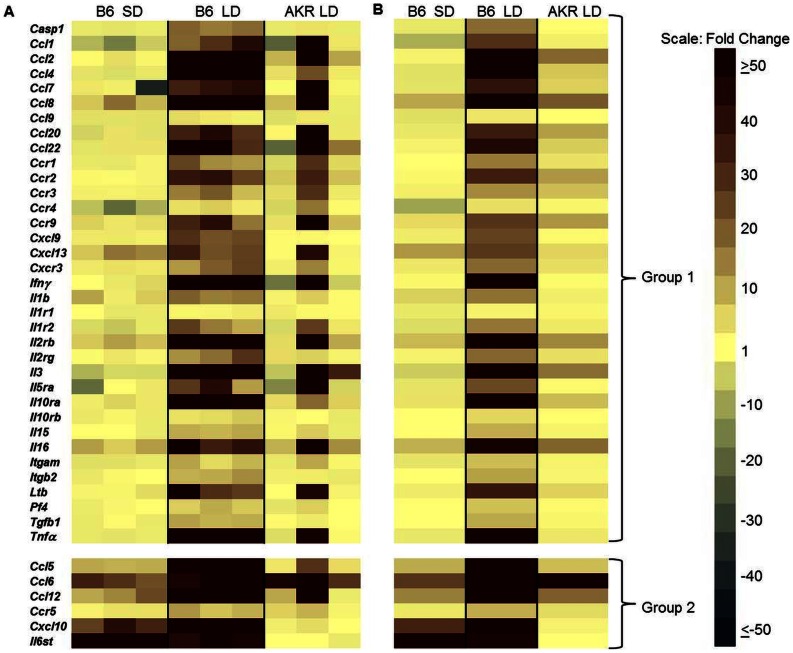
Livers of LD fed B6 mice display a proinflammatory cytokine profile. (A) Expression levels of the indicated genes in livers from individual mice relative to SD fed AKR mice (n = 3 per group). (B) Averaged values for each strain relative to AKR mice fed SD. Group 1 genes are significantly upregulated in B6 LD only; Group 2 genes are significantly upregulated in both B6 LD and B6 SD groups. A significant change is defined as ≥3 fold difference with P<0.05 relative to SD AKR controls.

To determine if there were specific immune function genes that were associated with hepatitis susceptibility, a pathway focused quantitative PCR based array was performed on livers from susceptible (B6) and resistant (AKR) mice. LD fed B6 mice significantly upregulated (P<0.05, 3 fold or greater change) 41 out of 84 inflammatory cytokines, chemokines and ligands included in the array (Fig. 4; Group 1 are genes exhibiting significant changes in LD fed B6 mice and Group 2 are genes exhibiting significant changes in both SD fed B6 and LD fed B6 mice compared to SD fed AKR mice). Of note, LD fed B6 mice upregulated numerous chemokines associated with the recruitment of CCR2 positive cells (CCL2, CCL7, CCL8 and CCL12; Fig. 4; Group 1). In the case of CCL12, this ligand was upregulated significantly (P<0.05, 3 fold or greater change) in B6 SD fed mice compared to SD fed AKR mice (Fig. 4; Group 2) indicating that even in the basal state this chemokine is more highly expressed in B6 mice. In addition, LD fed B6 mice upregulated several potent pro-inflammatory cytokines including IFNγ, IL-1β, and TNF-α. In contrast, in LD fed AKR mice there were no significant changes in inflammatory genes compared to SD controls (Fig. 4, Fig. S2). In some instances, in LD fed AKR mice the mean fold change (Fig. 4B) appeared quite large (e.g. CCL6) but the results were highly variable (Fig. 4A) and failed to achieve statistical significance compared to SD fed AKR controls. There were also a large number of cytokines which were not significantly altered in any groups of mice fed the LD compared to SD fed AKR controls (Fig. S2).

### CCR2 and CD44 Knockout Mice are Protected from Hepatitis Despite Histological Evidence of Steatosis and Elevated Hepatic Serum Enzymes

**Figure 5:**
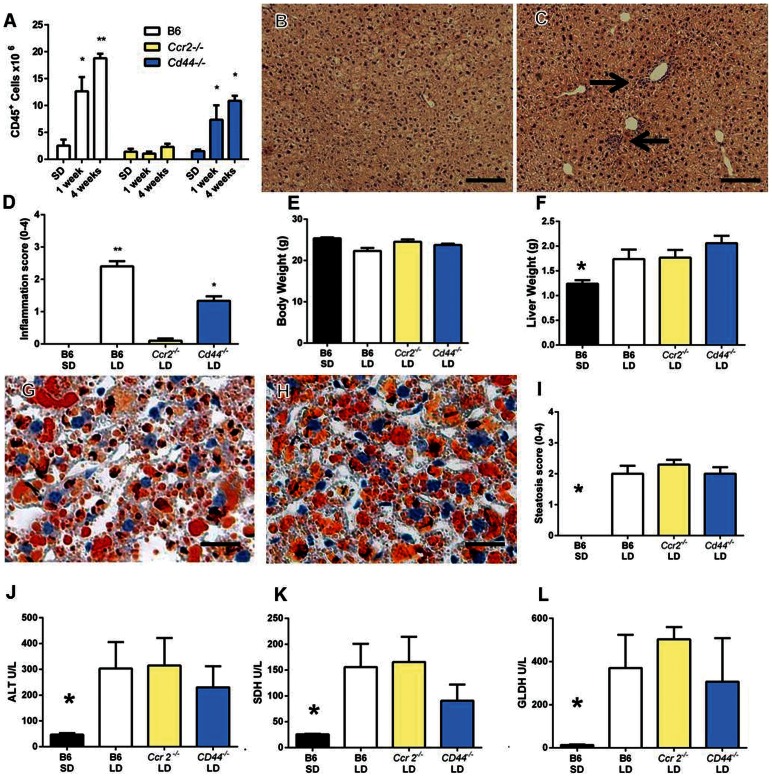
*Ccr2^−/−^* and *Cd44^−/−^* mice are resistant to hepatitis during LD feeding despite lipid accumulation and elevated serum enzymes. (A) Infiltration of CD45^+^ inflammatory cells into the livers of B6, *Ccr2^−/−^*, and *Cd44^−/−^* at one and four weeks of LD or SD feeding (*P<0.05 compared to SD controls, **P<0.05 compared to all groups, n = 3–6 per group). (B and C) Respective low power images from *Ccr2^−/−^* and *Cd44^−/−^* mice fed the LD for four weeks. In C, arrows point to inflammatory infiltrates restricted to large liver vessels. (D) Histological scoring of mice confirms moderate to severe inflammation in B6 LD mice and mild to moderate inflammation in *Cd44^−/−^* mice (**P<0.05 compared to all groups, *P<0.05 compared to SD fed or *Ccr2−/−* LD fed mice; scores represent means of n = 6–12 mice per group). (E) Lithogenic diet feeding did not induce obesity in any genotype. (F) Lithogenic diet feeding induced significant hepatomegaly in all strains (*P<0.05 compared to all other mice, n = 4–6 per group). (G–H) Oil-red-o staining of livers from *Ccr2^−/−^*(G), and *Cd44^−/−^* (H) mice demonstrate an accumulation of excess lipid irrespective of genotype. (I) Steatosis scoring demonstrates a significant increase in steatosis in all genotypes in response to LD feeding for 4 weeks compared to SD fed mice (**P<0.05 compared to all groups n = 6–10 mice per group). (J–L) Serum levels of hepatic enzymes are elevated in LD fed mice irrespective of genotype. (J and K, *P<0.05 against all LD groups; L, *P<0.05 compared to LD fed B6 and *Ccr2^−/−^* mice; n = 4–5 per group). B and C, bar represents 100µm; G–H, bar represents 20µm.

**Figure 6:**
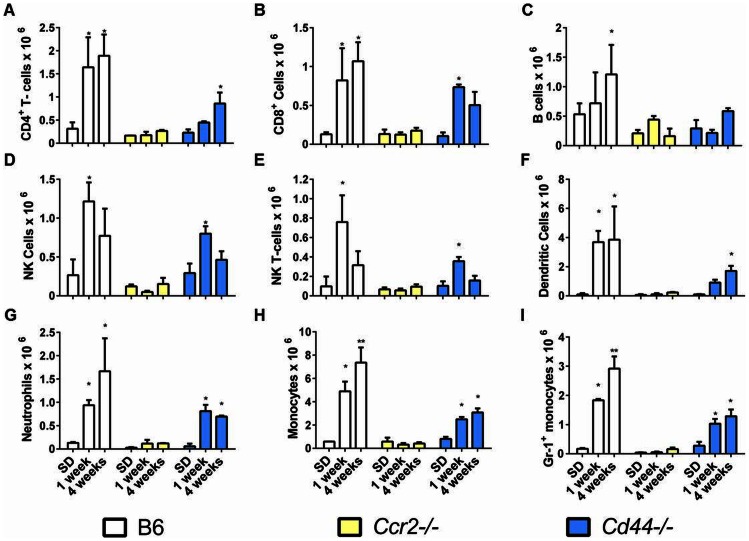
*Ccr2^−/−^* and *Cd44^−/−^* mice display altered inflammatory cell recruitment during LD feeding. (A–I) Composition of Liver Leukocytes in mice fed SD or LD (*P<0.05 compared to SD controls; **P<0.05 compared to all other groups). Cells are identified as in figure legend 2.

Because CCR2 and its ligands are upregulated in B6 mice and because CD44 expression is increased in LD fed B6 mice we wanted to determine if these two molecules were critical for hepatitis formation in this model. Therefore, we analyzed whether CCR2 and CD44 gene deleted mice were resistant to LD induced hepatitis. In contrast to B6 animals, *Ccr2^−/−^* mice displayed a striking absence of inflammatory infiltration either early (1 week) or later (4 weeks) during LD feeding (Fig. 5A). *Cd44^−/−^* mice displayed significant hepatitis at 1 and 4 weeks when compared to SD fed controls. However, inflammatory infiltration at 4 weeks was significantly less than B6 mice at the same time-point (Fig.5A). In contrast, hepatitis rapidly developed in B6 mice and significantly increased between 1 and 4 weeks of LD feeding (Fig. 5A). We asked whether the defect in recruitment was associated with any particular cell type. After 4 weeks of LD feeding, *CD44^−/−^* mice recruited significantly fewer monocytes and Gr-1^+^ (Ly6G^+^) monocytes compared to B6 mice (Fig. 6 H–I). LD fed *Ccr2^−/−^* failed to appreciably recruit any of the cell types examined (Fig. 6). Histopathological assessment of livers at 4 weeks of diet feeding confirmed the differences noted by flow cytometry (Fig. 5. B, C, D). Specifically, while B6 mice developed a similar pattern of moderate to severe multifocal inflammation to that shown in Fig. 1B inflammatory foci were absent in *Ccr2^−/−^* mice (Fig. 5B, 5D). In *Cd44^−/−^* mice, scattered inflammatory foci were noted most prevalently around the large vessels of the liver (Fig. 5C, black arrows) and histopathological scoring confirmed mild to moderate inflammation consistent with flow-cytometry data (Fig. 5D).

Gross examination of the mice after 4 weeks of feeding demonstrated that LD consumption did not promote obesity as indicated by similar weights compared to SD fed control animals (Fig. 5E). However, all mice fed LD displayed significant increases in liver weights compared to SD fed B6 mice consistent with hepatomegaly due to hepatic lipid accumulation (Fig. 5E). Livers of B6 (Fig. 5I), *Ccr2^−/−^* (Fig. 5G, I) and *Cd44^−/−^* mice (Fig. 5H, I) each developed significant and equivalent histological evidence of moderate steatosis as demonstrated by oil-red-O staining and histopathological scoring. Further, all LD fed groups, regardless of hepatitis protection or resistance, displayed significant serological elevations in hepatic enzymes indicative of hepatocellular damage (Fig. 5 J–L). Taken together, these data indicate that *Ccr2^−/−^* are completely protected and *Cd44^−/−^* mice are partially protected from hepatic inflammation despite similar histological evidence of steatosis and serological evidence of hepatocellular damage when compared to B6 mice.

### CD11b^+^ CCR2^+^ Cells Recruited to the Liver Display an Inflammatory Monocyte Phenotype

**Figure 7:**
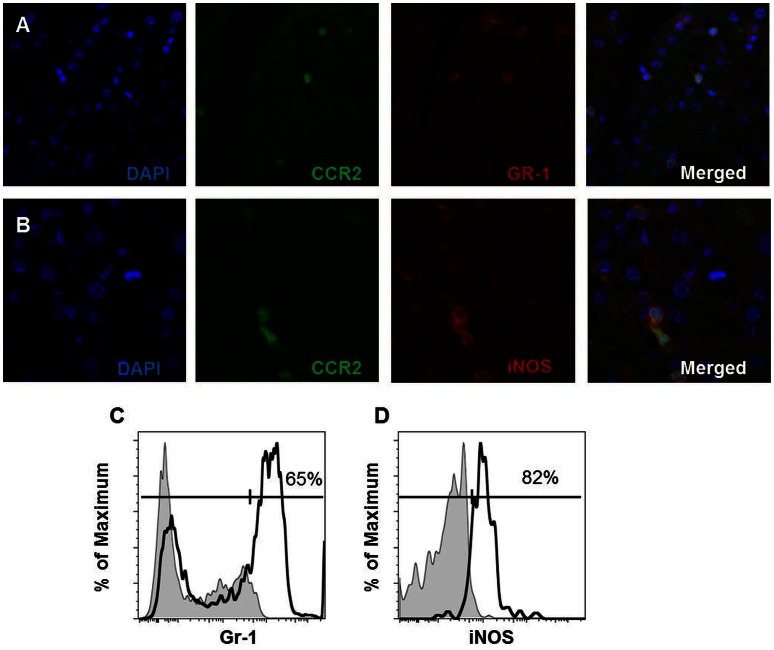
Monocytes in the liver of LD fed mice display an inflammatory phenotype. Co-expression of CCR2 and Gr-1 (A), as well as CCR2 and iNOS (B) in livers of CCR2 reporter mice. Flow cytometric analysis of hepatic CCR2^+^, CD11b^+^, Ly6C^−^ cells (solid line) confirms increase expression of Gr-1 (C) and iNOS (D) on CCR2^+^, CD11b^+^ cells compared to gray shaded regions which represent CCR2^+^, CD11b^−−^ populations stained for the same markers.

To further characterize the CCR2^+^ population of recruited monocytes we performed staining for Gr-1 (Ly6G) and iNOS, molecules associated with inflammatory monocytes, in the livers of CCR2 reporter mice fed the LD for four weeks. The majority of CCR2^+^, CD11b^+^ monocytes recruited to the liver were Gr-1^+^ (Fig. 7 A and C). Additionally, these cells expressed iNOS (Fig. 7 B and D). These findings are consistent with the marked increases in Gr-1+ monocytes noted in B6 but not *Ccr2^−/−^* mice (Fig. 6 I). The monocytes recruited during LD feeding possess the hallmarks of inflammatory monocytes.

### CD44 Mediates Hyaluronic Acid Binding During LD Feeding

**Figure 8:**
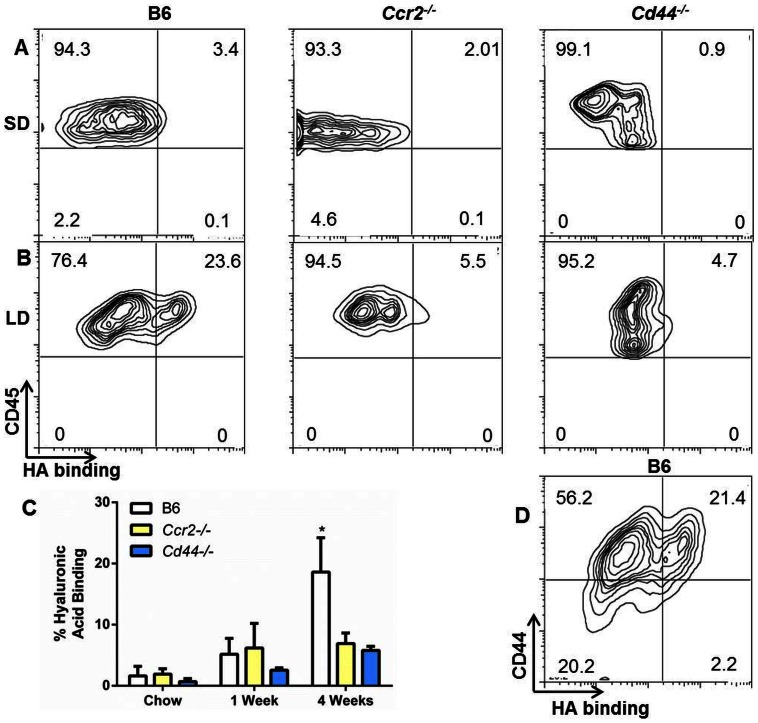
Hepatic leukocytes from B6 mice bind to hyaluronic acid in a CD44 dependent manner. (A) HA binding of CD45^+^ cells from a representative SD fed B6, *Ccr2^−/−^* and *Cd44^−/−^*mouse. (B) HA binding of CD45^+^ cells from a representative four week LD fed B6, *Ccr2^−/−^* and *Cd44^−/−^*mouse. (C) Cumulative HA binding results over time (n = 3–6 per group). (D) In CD45^+^ leukocytes from LD fed B6 mice, HA binding occurs on a subpopulation of CD44^+^ cells.

One of the mechanisms underlying recruitment of CD44^+^ cells to the liver involves CD44 binding to hyaluronic acid (HA) expressed on hepatic sinusoids [16]. However, not all forms of CD44 bind to HA [17]. Since *Cd44^−/−^* mice displayed diminished inflammatory infiltrates we wanted to determine if HA binding on hepatic leukocytes increased during LD feeding. Liver leukocytes of SD mice, regardless of genotype, did not bind appreciably to HA (Fig. 8A). LD feeding induced a population of HA binding liver leukocytes in B6 mice (Fig. 8B). In contrast, hepatic leukocytes of both *Ccr2^−/−^* and *Cd44^−/−^* mice remain HA binding negative after 4 weeks of LD feeding (Fig. 8B). Significant increases in HA binding were not present at 1 week of LD feeding but were seen at 4 weeks (Fig. 8C). HA binding did not occur appreciably on CD44^−^ cells and only occurred on a subset of CD44^+^ cells consistent with the fact that not all CD44 binds HA (Fig. 8D).

**Figure 9:**
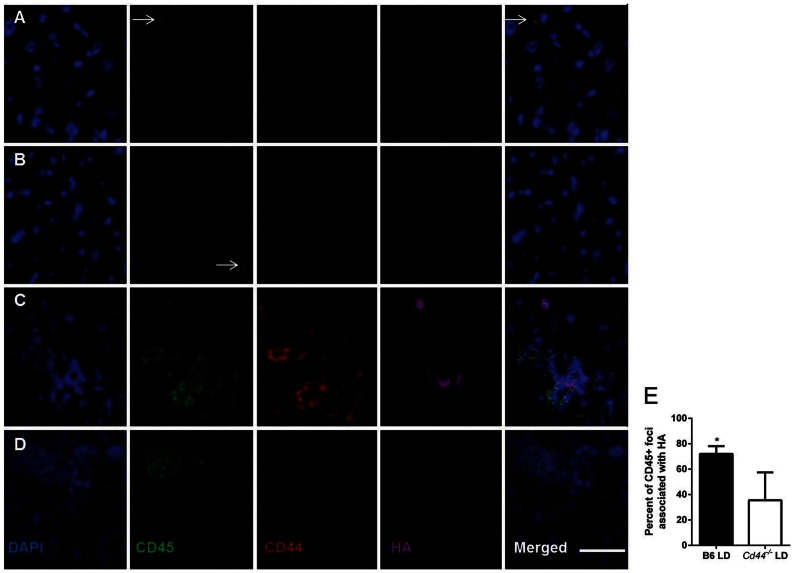
Leukocytes co-localize with HA in B6 diet fed mice. (A) B6 mice fed SD and (B) *Ccr2^−/−^* mice fed LD display rare CD45^+^ inflammatory cells (white arrows). (C) After 4 weeks of LD feeding B6 mice develop marked foci of CD45^+^ CD44^+^ cells that are often co-localized with HA expressing regions of the liver. (D) In contrast, *Cd44^−/−^* mice develop CD45^+^ inflammation without CD44 expression and decreased co-localization with HA. (E) Quantitative assessment of co-localization of HA with inflammatory foci demonstrates a high degree of co-localization in B6 mice and a moderate amount in *Cd44^−/−^* mice. CD45^+^ inflammatory foci were counted and scored positive if clusters of CD45^+^ cells occurred in direct contact with HA positive staining. Inflammatory foci were not identified in *Ccr2^−/−^* and SD fed mice and as a result they were not quantified. (*P<0.05, data represent counts analyzed by contingency tables and Fisher’s exact test from n = 4 mice per group).

Frozen sections of livers were next examined to determine if CD45^+^ cells co-localized in areas which were positive for HA. In B6 mice fed SD (Fig. 9A) or *Ccr2^−/−^* mice (Fig. 9B) few CD45^+^ cells were found, consistent with flow-cytometric and histological data. LD fed B6 mice displayed numerous CD45^+^ cells which were CD44^+^ and often associated with HA^+^ regions of the liver (Fig. 9C, 9E). In contrast, in *Cd44^−/−^* mice, CD45^+^ CD44^−^ areas were present but co localization with HA was reduced when compared to LD fed B6 mice (Fig. 9D, 9E). Taken together these data indicate that in LD fed B6 mice, CD45^+^CD44^+^ cells bind to HA and localize to HA positive areas.

### HA Binding Occurs Primarily on Monocytes, CD8^+^ T-cells and Dendritic Cells

**Figure 10:**
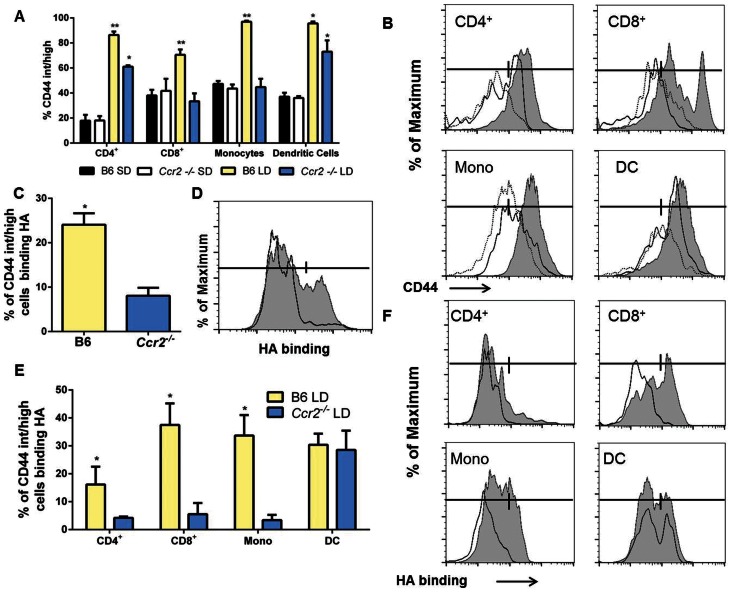
CCR2 deletion reduces HA binding and CD44 expression in T-cells and monocytes. (A) Binding of HA by liver leukocytes of LD fed B6 and *Ccr2^−/−^* mice. (A) Proportion of hepatic CD44^int/hi^ cells in SD and LD fed B6 and *Ccr2^−/−^* mice. LD fed B6 mice show significant increases in CD44^int/hi^ CD8^+^ cells, CD4^+^ cells, monocytes and dendritic cells when compared to SD fed control mice and significant increases in CD8^+^ cells, CD4^+^ cells, and monocytes when compared to LD fed *Ccr2^−/−^* mice (*:P<0.05 compared to SD fed group, **:P<0.05 compared to all groups). (B): Histograms showing CD44 expression in CD4^+^ cells, CD8^+^ cells, monocytes and dendritic cells from LD fed B6 mice (gray shaded region), LD fed *Ccr2^−/−^*mice (solid line) or SD fed B6 mice (dashed line) demonstrating an increase in CD44 expression in LD fed B6 mice compared to LD fed *Ccr2^−/−^* mice in all cells with the exception of dendritic cells. Bars represent gates for CD44^int/hi^ cells. (C) Binding of HA by CD44^ int/hi^ liver leukocytes of LD fed B6 and *Ccr2^−/−^* mice demonstrating increased HA binding in CD44^int/hi^ populations (*:P<0.05). (D) Representative histogram showing HA binding by CD44^ int/hi^ liver leukocytes from B6 (shaded) and *Ccr2^−/−^* (solid-line) mice. Bar represents gate used for positive and negative staining. (E) Percentage of HA binding of various CD44^int/hi^ leukocyte populations demonstrating significant increases in the binding of CD4^+^ cells, CD8^+^ cells and monocytes in B6 LD mice compared to *Ccr2^−/−^* LD mice. Both groups exhibited a high degree of HA positive binding in dendritic cells (*:P<0.05). Representative histograms showing HA binding by various populations of CD44^ int/hi^ liver leukocytes from B6 (shaded) and *Ccr2^−/−^* (solid-line) mice. Bar represents gate used for positive and negative staining for each cell type. For individual cell-types gating was determined by the background HA staining of a non-target group of CD44^−/low^ expressing cells.

The lack of HA binding by hepatic leukocytes from *Ccr2^−/−^* mice during LD feeding was an unexpected finding so we next examined if this was the result of altered CD44 expression or a difference in the binding capacity of the CD44 molecules. In response to LD feeding wild-type mice displayed increased numbers of CD44 intermediate (int) and high (hi) expressing CD4^+^, CD8^+^, monocytes, and dendritic cells (Fig. 10A and B; shaded region = LD fed B6 mice, solid line = LD fed *Ccr2^−/−^* mice, dashed line = SD fed B6 mice). In contrast LD fed *Ccr2^−/−^* mice displayed significantly fewer CD44^int/hi^ expressing CD4^+^, CD8^+^ and monocytes and a lack of any CD44^hi^ CD8^+^ T-cells (Fig. 10A and B; solid black line). When compared to SD fed mice, LD fed *Ccr2^−/−^* mice demonstrated significantly increased numbers of CD44^int/hi^ CD4^+^ and dendritic cells (Fig. 10A and B; solid black line). The surface expression of CD44 on other cell-types examined was not altered by diet or strain (Fig. S3A).

Next we examined the ability of CD44^int/hi^ cells to bind to HA. When CD45^+^CD44^int/hi^ expressing cells were exclusively examined B6 mice displayed greater HA binding when compared to *Ccr2^−/−^* mice (Fig. 10 C and D; B6: shaded region, *Ccr2^−/−^*: solid line). When specific CD44^int/hi^ cell-types were examined significantly increased HA binding was noted on CD8^+^ cells and monocytes from B6 mice compared to *Ccr2^−/−^* mice (Fig. 10 E and F; B6: shaded region, *Ccr2^−/−^*: solid line). With regard to CD4^+^ cells, HA binding was relatively low in both B6 and *Ccr2^−/−^* mice but nevertheless there was a significant difference between these 2 groups (Fig. 10 E and F; B6: shaded region, *Ccr2^−/−^*: solid line). Binding of HA on dendritic cells did not differ between the groups but was elevated when compared to other cell types examined (Fig. 10 E and F; B6: shaded region, *Ccr2^−/−^*: solid line, Fig. S3B). Other cell types displayed low and highly variable levels of HA binding (Fig. S3B). These data demonstrate that CD44 expression in response to LD feeding occurs primarily on T-cells, monocytes and dendritic cells. This induction is diminished in *Ccr2^−/−^* mice on all but dendritic cells. HA binding occurs primarily on CD8^+^ T-cells, monocytes and dendritic cells and *Ccr2^−/−^* mice fail to induce HA binding in both monocytes and CD8 T-cells.

### CCR2 Deficiency Prevents Hepatic Proinflammatory Cytokine Production

**Figure 11:**
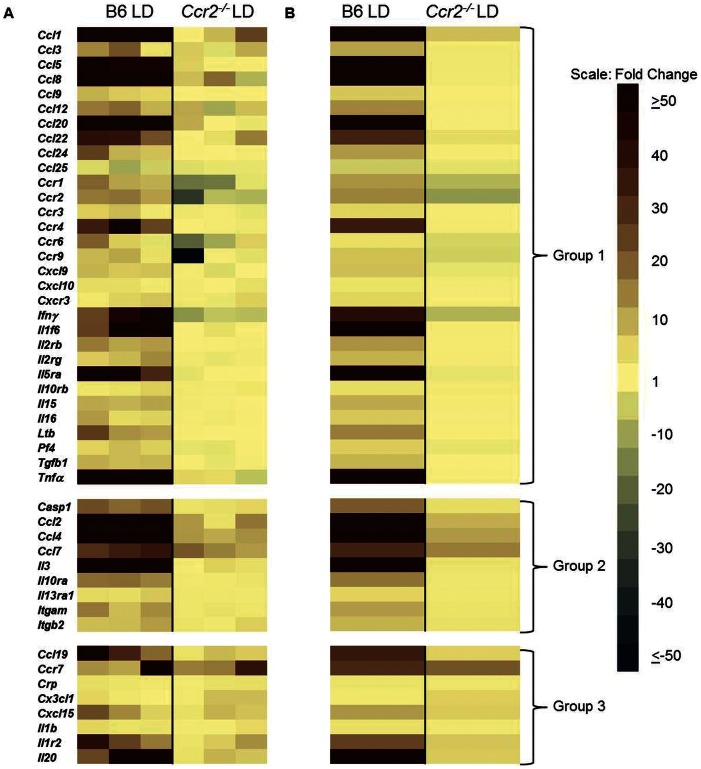
Livers of LD fed *Ccr2^−/−^* mice fail to develop the proinflammatory cytokine response of LD fed B6 mice. (A) Gene transcript levels in livers of individual LD fed B6 and *Ccr2^−/−^* mice relative to B6 SD. (B) Mean values of individual mice relative to B6 SD controls. Group 1 genes significantly altered in B6 LD only; Group 2 genes significantly altered in both B6 LD and *Ccr2^−/−^* LD groups and B6 LD mice are significantly increased over *Ccr2^−/−^* LD mice; Group 3 genes significantly altered in B6 LD and *Ccr2^−/−^* LD groups. A significant change is defined as ≥3 fold difference with P<0.05 relative to SD B6 controls (n = 3 per group; LD fed *Ccr2^−/−^* mice were compared to the previously described cohort, Fig. 4, of SD and LD fed B6 mice).

To determine if elimination of cellular infiltrates in *Ccr2^−/−^* mice results in an altered local cytokine/chemokine profile we performed focused pathway arrays on *Ccr2^−/−^* mice. Overall, *Ccr2^−/−^* mouse livers failed to display the proinflammatory signature of their wild-type counterparts which we previously reported (Fig. 11A individual mice, Fig. 11B mean values). There were three distinct groups of changes which occurred. A subset of genes was significantly changed in only LD fed B6 mice compared to SD fed B6 mice (Group 1) while a second subset was significantly upregulated in both LD fed groups with LD fed B6 mice still exhibiting significantly elevated expression levels compared to LD fed *Ccr2^−/−^* mice (Group 2). A third group of genes was upregulated and statistically indistinguishable between LD fed B6 and *Ccr2^−/−^* mice. Finally, there was a subset of genes which was not significantly altered in any of the experimental groups (Fig. S4). Overall, B6 mice fed the LD developed significant (P<0.05, +/−3 fold or greater change) alterations in 49 of 84 genes examined (Groups 1, 2 and 3) when compared to SD fed B6 controls (Fig. 11). In contrast, *Ccr2^−/−^* mice significantly altered only 17 of 84 analyzed genes and only 8 total genes were expressed at statistically equivalent levels to B6 LD fed mice (Fig. 11, Group 3). Overall, *Ccr2^−/−^* mice were protected from the production of deleterious pro-inflammatory cytokines in the liver. Interestingly, *Tnf-α* is markedly upregulated (>50 fold) in LD fed B6 mice. This cytokine is a critical inducer of CD44 upregulation and HA binding in both monocytes and T-cells consistent with the altered HA binding exhibited in these cell types in LD fed B6 but not *Ccr2^−/−^* mice [18], [19].

### CCR2 and CD44 Promote Stellate Cell Activation and Fibrosis During Hepatic Lipid Accumulation

**Figure 12:**
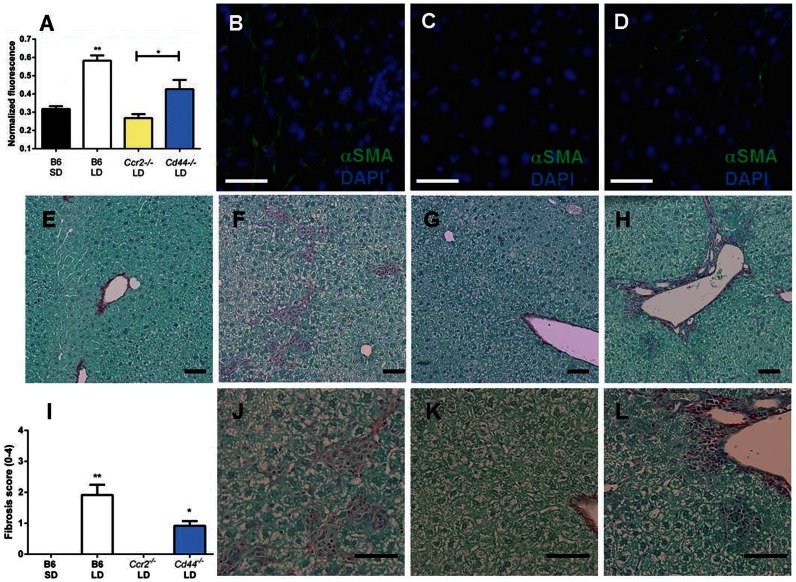
Livers from LD fed B6 mice display activated stellate cells and fibrotic lesions. (A) Liver tissue sections were stained with α-smooth muscle actin antibody (αSMA) and cell nuclei were stained with DAPI. α-smooth muscle actin signal (green fluorescence) was normalized to cell number (blue fluorescence) (n = 4 mice per group, 5 tissue sections per mouse; *P<0.05 compared to *Ccr2^−/−^* mice; **P<0.05 compared to all groups). A representative section from LD fed B6 mice (B), *Ccr2^−/−^* mice (C) and *Cd44^−/−^* mice (D). (E–H) Low and (J–L) high power magnification of pico-sirius stained SD fed B6 mice (E), LD fed B6 mice (F,J), *Ccr2^−/−^* mice (G,K) and *Cd44^−/−^* mice (H,L). (I) Fibrosis score was determined by sirius-red positive staining (**P<0.05 compared to all groups, *P<0.05 compared to B6 SD and *Ccr2^−/−^* LD). In E–H and J–L the bar corresponds to 25µm.

Since inflammatory mediators are capable of promoting fibrosis we wanted to determine if *Cd44^−/−^* and *Ccr2^−/−^* mice are protected from this pathology during LD feeding. We evaluated expression of smooth muscle actin, a marker of stellate cell activation, and we performed pico-sirius red staining to evaluate for the presence of fibrosis. B6 mice displayed significantly more activated stellate cells compared to all other groups of mice as evidenced by increased normalized smooth muscle actin expression (Fig. 12 A, B). In contrast, *Ccr2^−/−^* mice fed LD displayed levels of smooth muscle actin staining which were nearly identical to SD fed mice and were not noticeably evident by immunofluorescence (Fig. 12 A, C). *Cd44^−/−^* mice had a moderate, yet statistically insignificant, elevation in smooth muscle acting staining when compared to SD fed B6 mice; however, levels were statistically increased over *Ccr2^−/−^* mice fed the LD (Fig. 12 A, D).

Consistent with smooth muscle actin staining, SD fed B6 mice developed no fibrosis on pico-sirius red staining (Fig. 12 E, I) and livers from LD fed B6 mice had significant increases in fibrosis compared to all other groups (Fig. 12 F, J, I). *Ccr2^−/−^* mice did not display areas of positive fibrosis staining (Fig. 12 G, K, I) and *Cd44^−/−^* mice developed sporadic areas of mild fibrosis primarily in perivascular regions in association with inflammatory infiltrates (Fig. 12 H, L). Fibrosis scores for *Cd44^−/−^* mice were significantly elevated when compared to *Ccr2^−/−^* and SD fed B6 mice (Fig. 12I). Taken together, these data indicate that LD fed *Ccr2^−/−^* mice are completely protected from fibrosis and *Cd44^−/−^* mice are partially protected.

## Discussion

Our data reveal the independent and overlapping roles of CCR2 and CD44 in development and progression of hepatic inflammation during fatty liver disease. Our findings reinforce studies in humans that have identified CCR2 and CD44 as being increased in NASH patients and they establish a possible link between these two molecules in lipid mediated hepatitis progression in so far as *Ccr2^−/−^* mice are largely protected from CD44 mediated HA binding [6], [7], [20]. The monocytes emerging in the liver during hepatitis progression bare the hallmarks of inflammatory monocytes as they display a CCR2^+^, Gr-1^+^ (Ly6G), iNOS^+^ phenotype. Further, livers from susceptible LD fed B6 mice when compared to SD fed B6 mice display pronounced increases in expression of IFNγ, TNF-α, CXCL9, CXCL10, and CCL20 which are each associated with inflammatory monocyte activation. Finally, susceptible B6 mice display a proinflammatory signature consistent with the recruitment of a broad range of inflammatory cells.

CCR2 is known to be a key molecule for the recruitment of T-cells, monocytes and dendritic cells so it is not surprising that these cells were reduced in *Ccr2^−/−^* mice [21]–[23]. Interestingly, *Ccr2^−/−^* mice demonstrate broad protection against the induction of chemokines responsible for the recruitment of other inflammatory cells. Specifically, B6, but not *Ccr2^−/−^* mice display marked increases in chemokine ligands, other than *Ccl2*, which are known to recruit monocytes, T-cells and dendritic cells (*Ccl1*, *Ccl3*, *Ccl4*, *Ccl7*, *Ccl8*), B-cells (*Ccl20*), neutrophils (*Ccl3/Mip1α*, and *Pf4/Cxcl4*), and NK and NK T-cells (*Ccl3, Ccl4, Ccl8*) [22], [24]–[26]. In this way, CCR2 deficiency seems to offer pleiotropic protection from LD mediated hepatic inflammatory cell recruitment.

Previous work by others demonstrated that deficiency of CCL2, a chemokine ligand of CCR2, did not prevent hepatitis in a methionine-choline deficient (MCD) mouse model of NASH [27]. More recently, a study demonstrated that inhibition of CCL2 with an L-enantiomeric RNA oligonucleotide prevented monocyte accumulation in mice fed a MCD chronically and those treated with carbon tetrachloride acutely [28]. Our findings demonstrate that several independent CCR2 ligands (CCL2, CCL7, CCL8 and CCL12) are upregulated during hepatic lipid accumulation. These multiple redundant pathways may, in part, explain the previous discrepancies in these publications. That is, if pharmacological inhibition also affected other similar CCR2 ligands this could explain why gene disruption of CCL2 did not prevent disease but pharmacological inhibition did.

CD44 has previously been shown to be elevated in human NASH patients [6], [20]. We found that *CD44^−/−^* mice were incompletely, yet significantly protected from inflammation during LD feeding. Further, we found elevations in CD44 expression on monocytes, T-cells, and dendritic cells in LD fed B6 mice. CD8^+^ T-cells displayed a mixed intermediate and high CD44 expressing phenotype. Others have described similar CD8^+^ CD44 intermediate subsets generated under sterile inflammatory conditions and have characterized them as an early memory T-cell which develops into a CD44^hi^ phenotype [29]. Although not critically examined here it seems probable that the mixed T-cell population in this study is similar to those previously described [29]. We demonstrated that HA binding of leukocytes was absent early during LD feeding (1 week) but significantly increased by 4 weeks in susceptible B6 mice. Furthermore, we observed co-localization between CD45^+^ cells and HA in liver tissue sections in susceptible mice. Co-localization data should be interpreted with caution because HA is a known component of the fibrotic response and we saw increased fibrosis in B6 LD mice particularly in areas around inflammatory infiltrates. Indeed even in *CD44^−/−^* mice there was a moderate amount of co-localization noted. Likewise, detection of HA by confocal microscopy was limited and only large aggregates of HA were readily detectable.

In *Cd44^−/−^* mice there was an increase in liver CD45^+^ cells after one week of LD feeding, but then CD45^+^ numbers stabilized. In contrast, B6 mice displayed both an early increase and a continued influx of CD45^+^ cells. Taken together, these data demonstrate that HA binding to CD44 may represent an important step in progression of inflammation associated with LD induced liver disease. There are several reasons why this interaction may promote inflammation. First, as previously mentioned, HA serves as a critical ligand for immune cell recruitment [18], [19], [30]. Additionally, irrespective of immune recruitment, HA binding to CD44 induces a pronounced proinflammatory response which will further promote the recruitment of leukocytes [31].

Interestingly, *Ccr2^−/−^* mice also displayed decreased HA binding which appeared to occur primarily because of a lack of induction of the HA binding isoform of CD44. On inspection of hepatic expression data this finding is consistent with the known biological regulation of CD44 and HA binding. Specifically, we noted a marked (>50 fold) increase in *Tnf-α* expression in LD fed B6 mice which was absent in *Ccr2^−/−^* mice. TNF-α stimulates CD44 expression on T-cells and monocytes and TNF-α is the primary cytokine responsible for the production of the HA binding variant of CD44 on both T-cells and monocytes [18], [19], [32]. *Ifn*-γ which is also upregulated in B6 but not *Ccr2^−/−^* mice has also been shown to enhance TNF-α mediated binding of CD44 to HA [33]. It therefore seems reasonable to hypothesize that increased HA binding in B6 but not *Ccr2^−/−^* mice is the result of the markedly increased *Tnf-α* and *Ifn-γ* expression in B6 mice. Interestingly, HA binding will then feed forward on this cycle by inducing greater active CD44 expression and greater cytokine and chemokine production including the production of more CCL2 [31]. Dendritic cells of *Ccr2^−/−^* mice fed the LD bound to HA; however, the overall number of dendritic cells in these mice is still markedly reduced consistent with the known role of CCR2 in their recruitment [23], [34]. The exact mechanism of induction of HA binding in dendritic cells of *Ccr2^−/−^* mice is unknown but *Ccr2^−/−^* mice did develop mild yet statistically significant increases in both *Il-3* and *Il-1B* which are two cytokines known to promote HA binding [18], [30]. Alternatively, HA binding is a phenotypic occurrence during dendritic cell maturation so this phenotype may indicate that dendritic cells in the livers of LD fed mice, are displaying a maturation phenotype [35]. There is strong evidence that TLR stimulation of resident leukocytes occurs during steatosis and *Tlr4* is known to induce dendritic cell maturation so it follows that this may be involved in this phenotypic conversion [36], [37]. Recent evidence also suggests that lipid rich dendritic cells represent a distinct pro-inflammatory cell population in the livers of mice and humans [38]. It is therefore intriguing to speculate that dietary lipid loading of hepatic dendritic cells may also play a role in induction of this HA binding phenotype.

In addition to resistance to hepatitis, *Ccr2^−/−^* mice were also completely protected from stellate cell activation and development of hepatic fibrosis. These findings are consistent with acute carbon tetrachloride mouse models which demonstrate that CCR2^+^, Gr-1^+^, iNOS^+^monocytes are important for the induction of fibrosis [39]. Increased expression of the fibrotic markers TGFβ, CCL7 and PF4 and the abundant expression of Th1 cytokines in B6 mice compared to *Ccr2^−/−^* is also consistent with the fibrosis protected phenotype [39]. In contrast, *Cd44^−/−^* mice displayed an intermediate phenotype with regard to stellate cell activation and fibrosis. Stellate cell activation and fibrosis appeared dependent upon the presence of inflammatory cell infiltrates, because in areas of the liver lacking inflammatory foci, there was little fibrosis noted even in susceptible strains. Others have demonstrated that osteopontin, a ligand of CD44, is important for promoting fibrosis and promotion of NAFLD/NASH [40], [41]. The osteopontin gene *Spp1* was present in our pathway array but was not significantly altered by LD at 4 weeks of feeding in either susceptible or resistant strains. In an MCD model of NAFLD others have demonstrated that increased hepatic osteopontin expression was not detected until 4 weeks of MCD diet feeding [42]. Once this induction occurred it remained high throughout the study. Therefore, the lack of induction of the osteopontin gene in our study may merely indicate that gene expression was analyzed prior to the onset of detectable osteopontin production. We therefore, cannot rule out the possibility that osteopontin also played a role in the protection of *Cd44^−/−^* mice in this model. Indeed based upon other models and data in humans the role of osteopontin in disease progression is convincing [40]–[42]. Our data do however describe an alternative and perhaps complementary proinflammatory mechanism of CD44 mediated hepatic inflammation through activation of hyaluronic acid binding.

Our data are consistent with a recent study using a choline deficient mouse model of NAFLD/NASH which demonstrated a critical role for CCR2 in disease pathogenesis [15]. In that study, *Ccr2^−/−^* mice displayed significant decreases in steatosis and liver recruitment of inflammatory monocytes. The authors also demonstrated that TLR4 and TLR9 signaling in Kupffer cells initiated *Ccl2* production [15]. Our results support and extend their study in a different model of hepatic lipid accumulation because we show that failure to recruit monocytes leads to a defect in recruitment of a variety of cell-types and a widespread reduction of a proinflammatory cytokine and chemokine production consistent with the role of CCR2 in the recruitment of other inflammatory cells [14], [21], [43]. We also demonstrated an inhibition of CD44-HA binding predominantly in monocytes and CD8^+^ T-cells of *Ccr2^−/−^* mice thereby linking CD44 recruitment and CCR2. In the choline deficient model, *Ccr2^−/−^* mice were significantly protected from inflammation, lipid accumulation and serum elevations in ALT [15]. Our data are also consistent with a recent publication which demonstrated that *Cd44^−/−^* mice fed a high-fat diet were protected from hepatic inflammation and inflammatory cytokines [44]. In that study, *Cd44^−/−^* mice were also protected from hepatic steatosis, displayed significantly smaller livers when compared to wild-type counterparts, and had significantly reduced serum liver enzyme values [44]. With regard to the steatosis protection demonstrated in these two studies but not in the current study, these differences are probably the result of the method of disease induction. Specifically, LD feeding delivers lipid to the hepatobiliary system largely through chylomicron remnants and occurs shortly after ingestion [45]–[47]. Obesity and deposition of lipid in visceral adipose tissue does not occur, likewise insulin resistance does not develop [48], [49]. Once in the liver, fatty acids from chylomicron remnant absorption may directly contribute to pathogenesis and cholesterol will induce triglyceride and fatty acid production through LXR responsive genes [50]–[52] Our data demonstrating steatosis without inflammation in *Ccr2^−/−^* and *Cd44^−/−^* mice are likely the result of the lipid source being directly related to dietary absorption thereby negating the role of the immune system on hepatic lipid accumulation. We did not specifically examine hepatic lipids biochemically and cannot rule out the possibility that there are biochemical differences in hepatic lipid content, but our data do demonstrate that *Ccr2^−/−^* and *Cd44^−/−^* mediated protection may occur independently of histological protection from steatosis.

Serological elevation of hepatic enzymes despite protection from inflammation was also a surprising finding in *Ccr2^−/−^* and *Cd44^−/−^* mice. However, these data are consistent with several studies analyzing human populations with NAFLD/NASH. That is, serum ALT poorly correlates with histological grade of inflammation and fibrosis [53]–[55]. Even when the same patients are followed over time, serial ALT level analysis fails to predict histological changes in NAFLD patients [54]. We have previously demonstrated in this model that lipid accumulation in hepatocytes leads to a marked increase in reactive oxygen species and oxidative DNA mediated damage [10]. It is well established that, oxidative stress of hepatocytes ultimately leads to cytosolic and mitochondrial membrane damage which may result in cell death by necrosis or apoptosis [56]. Based upon significant increases in histological steatosis score, and lipid induced hepatomegaly it seems likely that elevations in these enzymes are occurring at least in part as a result of lipid accumulation and oxidative mediated hepatocyte damage.

We hypothesize, based upon our data and others, that in lithogenic diet feeding, hepatic lipid accumulation leads to hepatocellular damage by lipid mediated oxidant products [10], [56]. *Ccl2* expression is then induced, in part through TLR stimulation of resident inflammatory cells [15], [37]. This in turn recruits CCR2 positive cells, primarily monocytes. These recruited cells produce additional proinflammatory cytokines and chemokines, thereby inducing the recruitment of other inflammatory cell-types. T-cells and macrophages increase CD44 expression and become HA binding in response to marked upregulation of TNFα. This binding promotes hepatic retention and induces additional proinflammatory cytokine and chemokine production. Cytokine production then contributes to stellate cell activation and hepatic fibrosis. Our data are an important step forward in understanding the contribution and interaction of the CCR2 and CD44 pathways in hepatic inflammation associated with hepatic lipid accumulation. Our study support and extend recent findings demonstrating the role of CCR2 and CD44 in inflammation during hepatic steatosis. This study demonstrates that pro-inflammatory cytokine production in the liver is mediated at least in part by CCR2^+^ cells because genetic deletion of CCR2 alleviates both cellular and molecular inflammation even in livers that are lipidotic. Furthermore, we show that CD44-HA binding occurs over the course of disease progression and that CCR2 deficiency partially eliminates this phenotype.

## Supporting Information

Figure S1 **Gating strategy and representative staining data from a B6 mouse fed the LD for 4 weeks.** Total liver preps were gated on FSC/SSC to exclude debris and cell clumps. Cells were gated based upon single staining of CD45^+^ cells and the FSC/SSC gate of the CD45^+^ cells was applied to all samples. Representative plot of C56BL/6 CD45^+^ cells after 4 weeks of LD showing CD11b/Ly6C staining.
(TIF)

Figure S2 **Transcript levels of a subset of genes remain unaltered.** (A) Expression levels of the indicated genes in livers from individual mice relative to SD fed AKR mice (n = 3 per group). (B) Averaged values for each strain relative to AKR mice fed a SD. Genes displayed did not achieve statistical significance. * *Xcr* was significantly elevated in SD fed B6 mice compared to SD fed AKR mice but data were unreliable because the gene was undetectable in SD AKR controls and weakly expressed in all groups.
(TIF)

Figure S3 **CD44 surface expression and HA binding are not significantly different in some cell-types.** (A) CD44 ^int/hi^ expressing B, NK, NK T-cells, and neutrophils remain unchanged regardless of diet or genotype. (B) HA binding phenotype of B-cells, NK cells, NK T-cells and neutrophils does not vary in LD fed *Ccr2^−/−^* and LD fed B6 mice. Neutrophils exhibit a minor HA binding phenotype and all other cells fail to bind to HA in response to LD feeding.
(TIF)

Figure S4 **Transcript levels of a subset of genes remain unaltered.** (A) Gene transcript levels in livers of individual LD fed B6 and *Ccr2^−/−^* mice relative to B6 SD. (B) Mean values of individual mice relative to B6 SD controls (n = 3 per group; LD fed *Ccr2^−/−^* mice were compared to the previously described cohort of SD and LD fed B6 mice).
(TIF)
