# Supplementary figures and images for: CCR2 and CD44 Promote Inflammatory Cell Recruitment during Fatty Liver Formation in a Lithogenic Diet Fed Mouse Model
Source: PLoS One. 2013 Jun 7;8(6):e65247. doi: 10.1371/journal.pone.0065247 (PMC3676479; doi:10.1371/journal.pone.0065247)

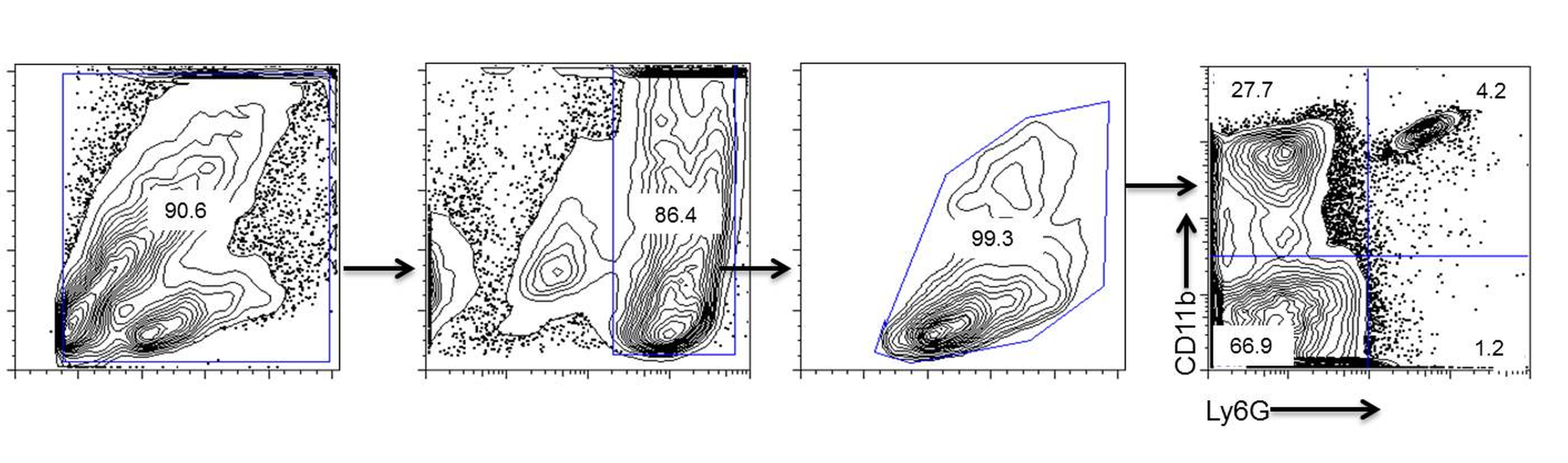

Supplement: Figure S1 — Gating strategy and representative staining data from a B6 mouse fed the LD for 4 weeks. Total liver preps were gated on FSC/SSC to exclude debris and cell clumps. Cells were gated based upon single staining of CD45+ cells and the FSC/SSC gate of the CD45+ cells was applied to all samples. Representative plot of C56BL/6 CD45+ cells after 4 weeks of LD showing CD11b/Ly6C staining. (TIF) [file pone.0065247.s001.tif]

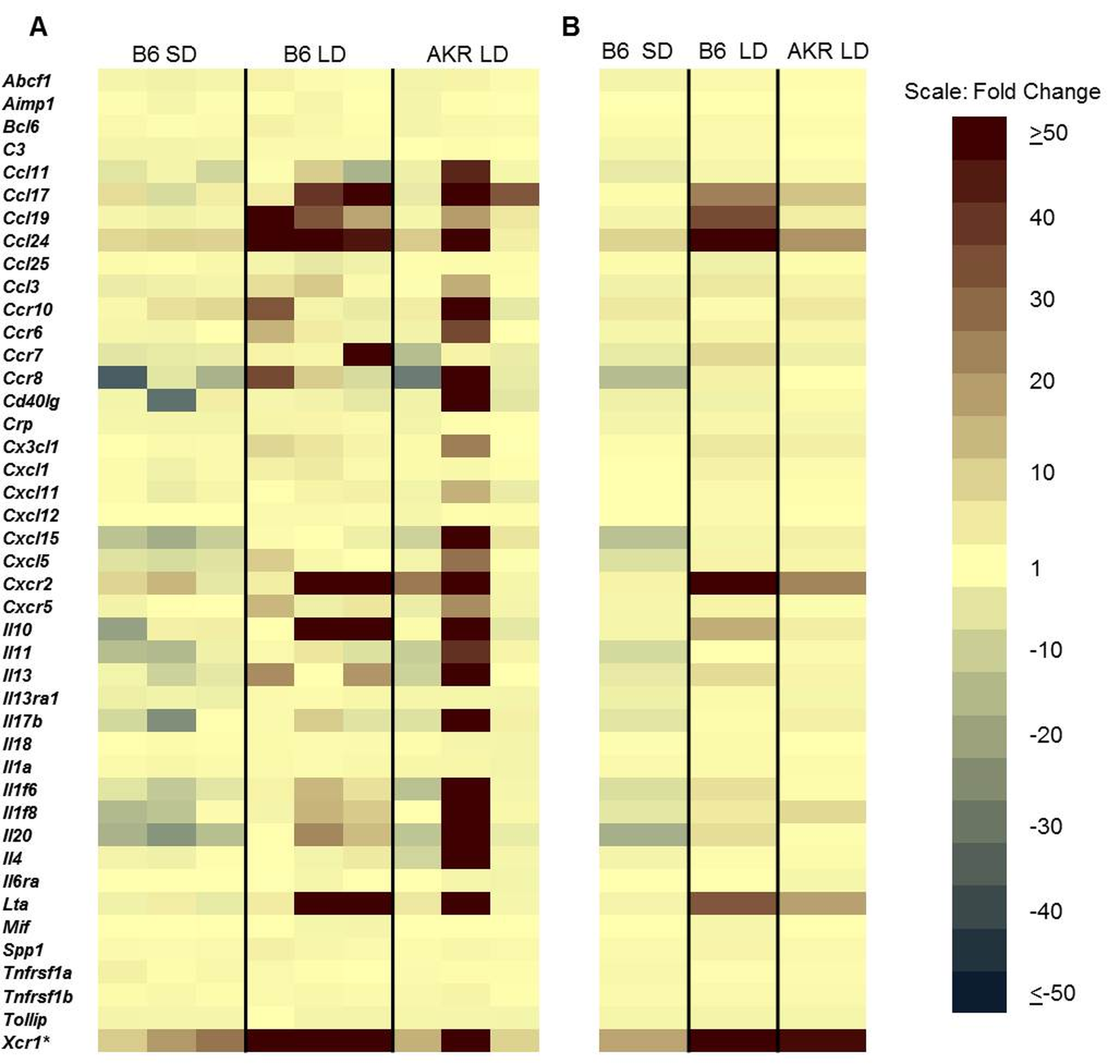

Supplement: Figure S2 — Transcript levels of a subset of genes remain unaltered. (A) Expression levels of the indicated genes in livers from individual mice relative to SD fed AKR mice (n = 3 per group). (B) Averaged values for each strain relative to AKR mice fed a SD. Genes displayed did not achieve statistical significance. * Xcr was significantly elevated in SD fed B6 mice compared to SD fed AKR mice but data were unreliable because the gene was undetectable in SD AKR controls and weakly expressed in all groups. (TIF) [file pone.0065247.s002.tif]

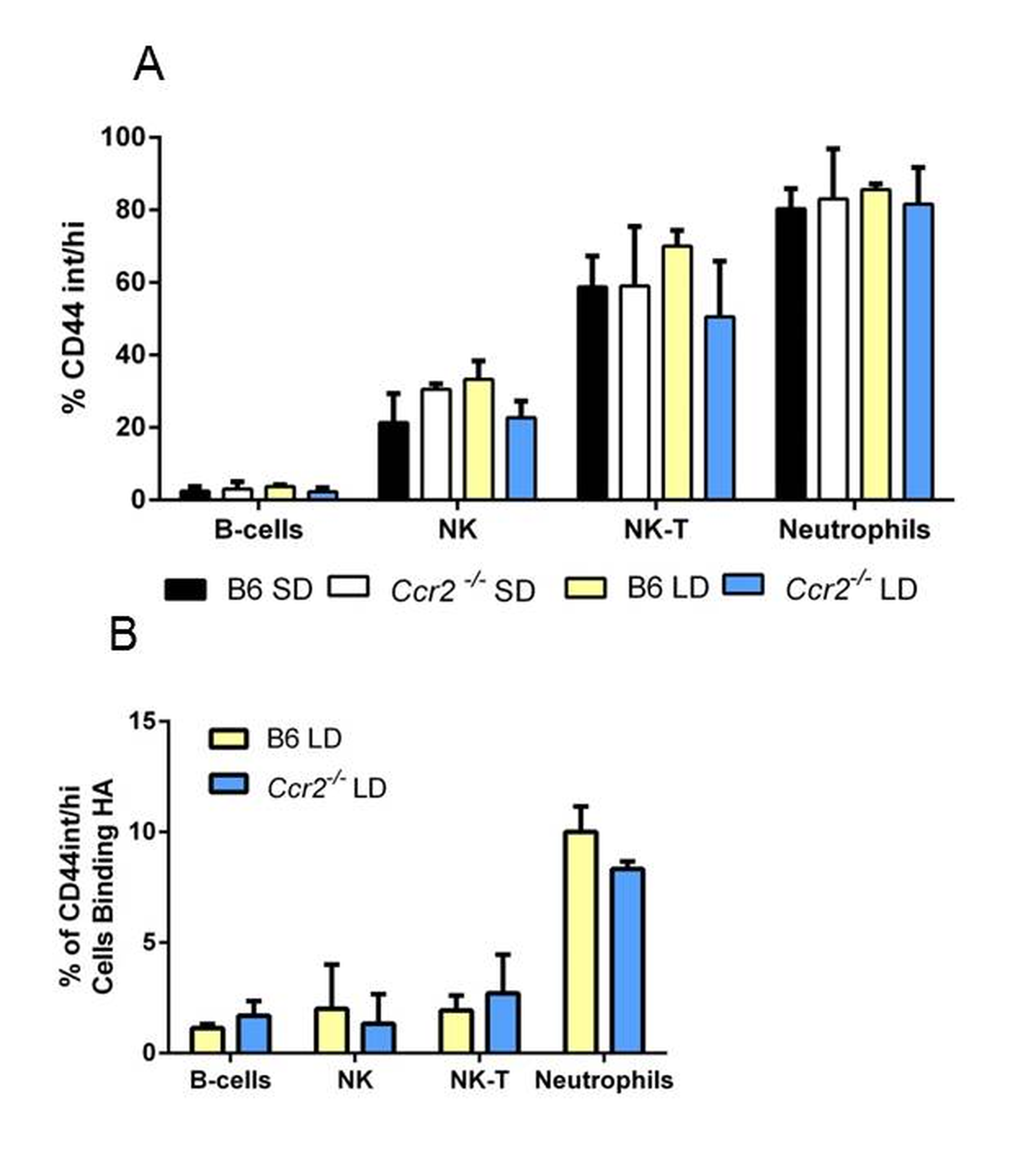

Supplement: Figure S3 — CD44 surface expression and HA binding are not significantly different in some cell-types. (A) CD44 int/hi expressing B, NK, NK T-cells, and neutrophils remain unchanged regardless of diet or genotype. (B) HA binding phenotype of B-cells, NK cells, NK T-cells and neutrophils does not vary in LD fed Ccr2−/− and LD fed B6 mice. Neutrophils exhibit a minor HA binding phenotype and all other cells fail to bind to HA in response to LD feeding. (TIF) [file pone.0065247.s003.tif]

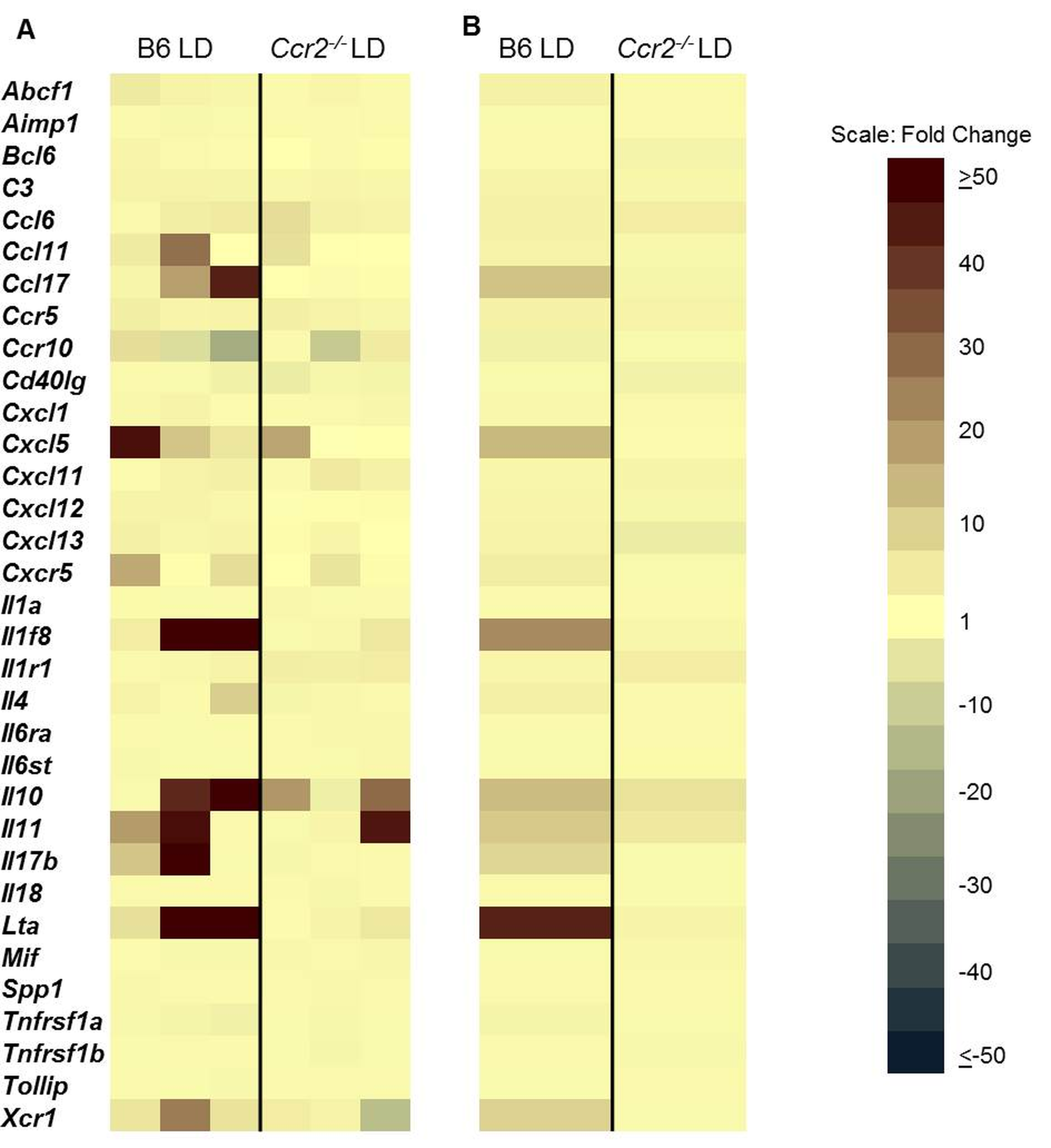

Supplement: Figure S4 — Transcript levels of a subset of genes remain unaltered. (A) Gene transcript levels in livers of individual LD fed B6 and Ccr2−/− mice relative to B6 SD. (B) Mean values of individual mice relative to B6 SD controls (n = 3 per group; LD fed Ccr2−/− mice were compared to the previously described cohort of SD and LD fed B6 mice). (TIF) [file pone.0065247.s004.tif]
